# A Comparative Morphological Study on the Characteristics of Egg Envelopes of Three Cultrinae Fishes (Cyprinidae, Teleostei) in Korea

**DOI:** 10.3390/life14070840

**Published:** 2024-07-01

**Authors:** Cheol-Woo Park, Jae-Goo Kim

**Affiliations:** 1 Alpha Research and Ecology Institute, Gunsan 54151, Republic of Korea; pcw0607@jbnu.ac.kr; 2 Department of Biological Science, College of Natural Sciences, Jeonbuk National University, Jeonju 54896, Republic of Korea

**Keywords:** zona radata, pore canals, non-structure, micropyle, Cultrinae

## Abstract

Three species of subfamily Cultrinae currently live in Korea, but *Erythroculter erythropterus* has been introduced into the Nakdonggang River and has taken over the habitat, reducing the habitat of *Culter brevicauda*. Only the endangered species *C. brevicauda* still lives in the Yeongsangang River, and it is necessary to be careful not to introduce *E. erythropterus* in the future. *Hemiculter eigenmanni* is also found throughout the country. In order to effectively manage and conserve the species in its various habitats and against invasions, this study was initiated. The ultrastructure of the egg envelopes of three species of Cultrinae inhabiting the Geumgang and Yeongsangang Rivers—*E. erythropterus*, *C. brevicauda*, and *H. eigenmanni*—were observed. It was found that the zona radiata of the egg envelopes of all three species were divided into two layers, an outer and inner layer, with the outer surface having a non-structural form. This form is characteristic of fishes with muddy, stagnant habitats or spawning grounds. The number of pore canals on the surface of the egg envelopes was 83 for *E. erythropterus*, 75 for *C. brevicauda*, and 58 for *H. eigenmanni* per 10 μm^2^, and the thickness was 7.89 ± 0.34 μm, 12.27 ± 0.46 μm, and 7.42 ± 0.24 μm, respectively. The shape of the micropyle demonstrated a funnel shape narrowing toward the inner diameter in all three species, and the size of the inner diameter was 6.62 ± 0.29 μm in *E. erythropterus*, 4.19 ± 0.39 μm in *C. brevicauda*, and 3.98 ± 0.46 μm in *H. eigenmanni*. The differences between species were identified in the number of pore canals, thickness, and micropyle inner diameter of egg envelopes, which were species-specific. Our study reveals a morphological mechanism in the egg envelope that prevents the formation of interspecific hybrids, and these features can be taxonomic traits that clarify species names. It also provides useful data for the production (breeding) of the second generation in aquaculture.

## 1. Introduction

The external morphology of the eggs of teleost fish varies between species, and these differences are used to create ecological and reproductive isolation. This function prevents the formation of hybrids between the different species. These differences are also important for distinguishing morphologically very similar species. In the recently developed aquaculture field, morphological studies of eggs are an important part of the development of superior species [1,2,3]. The eggs of teleosts are surrounded by a non-cellular egg envelope, the zona radiata (ZR), which is usually divided into two or three layers [4,5,6,7,8]. The inner layer is composed of a thick fibrous material, and the outer layer of a thin, highly electron-dense material [3,9]. These ZR serve to protect against physical impacts and chemical penetration from the external environment, and their structure and thickness are species-specific and influenced by habitat and spawning grounds [10,11,12].

The subfamily Cultrinae of the family Cyprinidae has approximately 18 genera and 80 species known worldwide, mostly inhabiting water systems in East Asia, including China, Mongolia, Russia, Taiwan, and Korea [13,14,15]. Four species, namely *Erythroculter erythropterus*, *Culter brevicauda*, *Hemiculter eigenmanni*, and *Hemiculter leucisculus*, from three genera, *Erythroculter*, *Culter*, and *Hemiculter*, have been reported to inhabit Korea [16,17]. *E. erythropterus* has recently been introduced to the Nakdonggang River and other rivers, showing a nationwide distribution, while *C. brevicauda* is only found in the Yeongsangang and Nakdonggang Rivers, but its population is declining as a result of the distribution of *E. erythropterus* and *H. eigenmanni* throughout Korea, and a very narrow range of populations living only in the estuaries of the Hangang River system is currently *H. leucisculus*. However, they are carnivorous in their feeding preferences, which can cause ecological disturbances when they leave their original habitats [17,18]. Since the recent introduction of *H. leucisculus* for aquaculture purposes in Central Asia along the Caspian Sea coast, its population has rapidly increased, causing significant effects on freshwater ecosystems [19,20,21]. In Korea, it has been reported that *E. erythropterus* and *Opsariichthys uncirostris amurensis* have invaded the Nakdonggang River from their original habitat, adversely affecting freshwater ecosystems, such as the largemouth bass *Micropterus salmoides* and bluegill *Lepomis macrochirus*, which are alien species [22]. Furthermore, in Korea, they are divided into two species (*E. erythropterus* and *C. brevicauda*) and are distinguished by morphological differences, such as carination on the abdomen and scale size. Internationally, they are not widely recognized. *E. erythropterus* is recognized in China and Taiwan under four different genera and scientific names, namely *Chanodichthys erythropterus*, *C. erythropteru*s, *E. erythropterus*, and *Culterichthys erythropterus*, and *C. brevicauda* is recognized as *C. alburnus*, *C. brevicauda*, and *E. adokii* [13,14,23,24,25]. Moreover, *C. brevicauda* has been protected in Korea because its population and habitat are declining. Therefore, although they are morphologically distinct, they are accepted as synonyms, and extensive taxonomic research is required. From these points of view, Cultrinae species are causing economic and ecological damage in Korea. Firstly, the anthropogenic introduction of *E. erythropterus* into the habitat of endangered *C. brevicauda* has reduced the population of *C. brevicauda* and established it as the top predator in each stream, much like largemouth bass. Secondly, there is still global confusion regarding the scientific names. This study aimed to investigate the preservation of *C. brevicauda* populations in the face of the spread of *E. erythropterus*. While *H. eigenmanni* is clearly distinguished by morphological differences, *E. erythropterus* and *C. brevicauda* are not yet taxonomically recognized (=synonym). Although the differences in morphology are minimal, they do exist and can be used as a basis to support this argument. Therefore, we compared their oocyte structures to identify differences between species.

## 2. Materials and Methods

**Figure 1:**
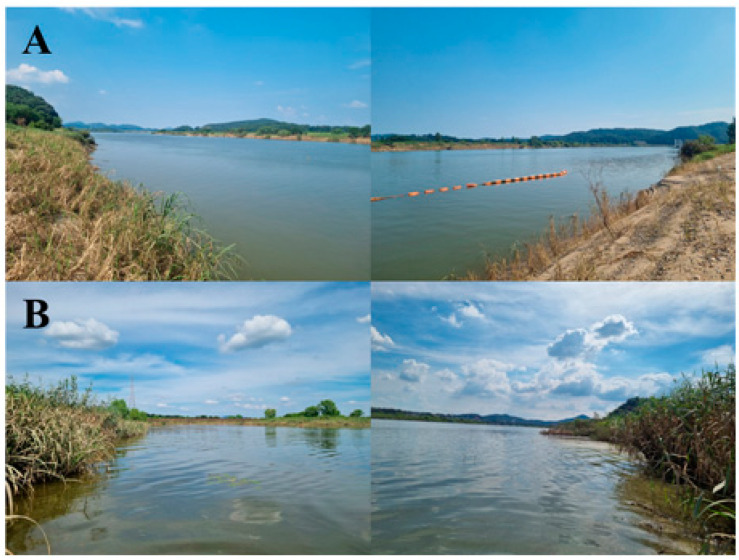
Sampling sites of three Cultrinae fishes in Korea: (**A**) Geumgang River; (**B**) Yeongsangang River.

Gravid females of three species were collected using a triangle net from the Geumgang and Yeongsangang Rivers from May to July 2022 (spawning season)—*E. erythropterus* (36°18′14″ N, 126°55′18″ E, Hoam-ri, Gyuam-myeon, Buyeo-gun, Chungcheongnam-do, Republic of Korea) in Geumgang River, and *Culter brevicauda* and *Hemiculter eigenmanni* (35°0″2′ N, 126°41″6′ E, Godong-ri, Geumcheon-myeon, Naju-si, Jeollanam-do, Republic of Korea) in Yeongsangang River (Figure 1). The bottom structures were modified according to the Cummins [26] method using mud (M: <0.1 mm), sand (S: 0.1–2 mm), pebble (P: 2–64 mm), cobble (C: 64–256 mm), boulder (B: >256 mm), and rock (R: large stone and bedrock), and the required ratio was obtained by visual examination. The permission to catch *C. brevicauda*, an endangered species, was granted by the Ministry of Environment, Republic of Korea (April 2022, license number: No. 2022-22). The study followed the Guide for the Care and Use of Laboratory Animals (2011), provided by the National Institutes of Health, USA. All experimental procedures were performed under the supervision of the Institutional Animal Care and Use Committee of the Chonbuk National University, Republic of Korea. All the experiments were performed under MS-222 anesthesia, and all efforts were made to minimize pain in the animals.

After anesthetization with MS-222, samples for light microscopy analysis were obtained by removing the ovaries and analyzing the most mature eggs among the cells in the ovarian cavity. Samples for electron microscopy and toluidine staining were obtained by artificially inseminating the most mature eggs released by pressing the abdomen of the fish with male sperm (*n* = 20). The extracted eggs were fixed in 10% neutral buffered formalin, dehydrated using a graded ethanol series (60–100%), and cleared in xylene. The samples were embedded in wax (Paraplast, Leica, Nußloch, Germany), and 5 μm sections were deparaffinized and stained with Harris’s hematoxylin and eosin [27]. For photographs and evaluation of the ZR, a light microscope (AX 10, Carl Zeiss, Jena, Germany) was used with AxioVision (LE REL 4.5, Carl Zeiss, Jena, Germany). The oocyte size utilized in each study method was measured by selecting a total of 20 mature eggs from all 3 species and measuring their diameter. For toluidine staining, fixed tissues were dehydrated, and blocks were prepared using an Epon mixture (Epon 812, EMS, Hatfield, PA, USA). Epon blocks were sectioned at 0.8 μm using an ultramicrotome (Reichert Ultracut S, Leica, Nußloch, Germany) and stained with 1% toluidine blue. The samples were examined using a light microscope AX10 and analyzed with AxioVision 4.5. 

For scanning electron microscopy (SEM), the fragments were fixed with 2.5% glutaraldehyde in 0.1 M phosphate buffer at pH 7.2. A post-fixation procedure was performed using 1.0% osmium tetroxide in the same buffer. After dehydration in a graded ethanol series (60–100%) and drying to a critical point using tert-butyl alcohol, the dried samples were coated with osmium tetroxide using a plasma coater (HPC-1SW, Vacuum Device Inc., Tokyo, Japan) and then filmed with an SEM instrument (S-300N, Hitachi, Tokyo, Japan) operating at 15 kV. For transmission electron microscopy (TEM), tissues that were fixed and dehydrated as described for the SEM were embedded in an Epon 812 mixture. The fragments were observed using a TEM device (H-7650, Hitachi, Tokyo, Japan) operating at 100 kV. The experimental values are expressed as the mean ± standard error of the mean. GraphPad Prism (version 9.0; GraphPad Inc., San Diego, CA, USA) and one-way analysis of variance (ANOVA) were used for multiple comparisons, followed by Dunnett’s test. Statistical significance was set at *p* < 0.001.

## 3. Results

Our analysis using the Cummins [26] method revealed that the substrate of the Geumgang River inhabited by *E. erythropterus* was composed of 80% sand, 10% mud, and 10% boulders, while the substrate of the Yeongsangang River inhabited by *C. brevicauda* and *H. eigenmanni* was more homogenous with 60% sand and 40% mud, and the habitats of the two rivers were relatively similar. As is typical of large rivers, the flow velocity is low (Figure 1). The oocyte sizes utilized in the analysis were 1.17 ± 0.09 mm for *E. erythropterus*, 1.08 ± 0.06 mm for *C. brevicauda*, and 0.79 ± 0.04 mm for *H. eigenmanni*. The egg size was similar between *E. erythropterus* and *C. brevicauda*, with *H. eigenmanni* being the smallest.

**Figure 2:**
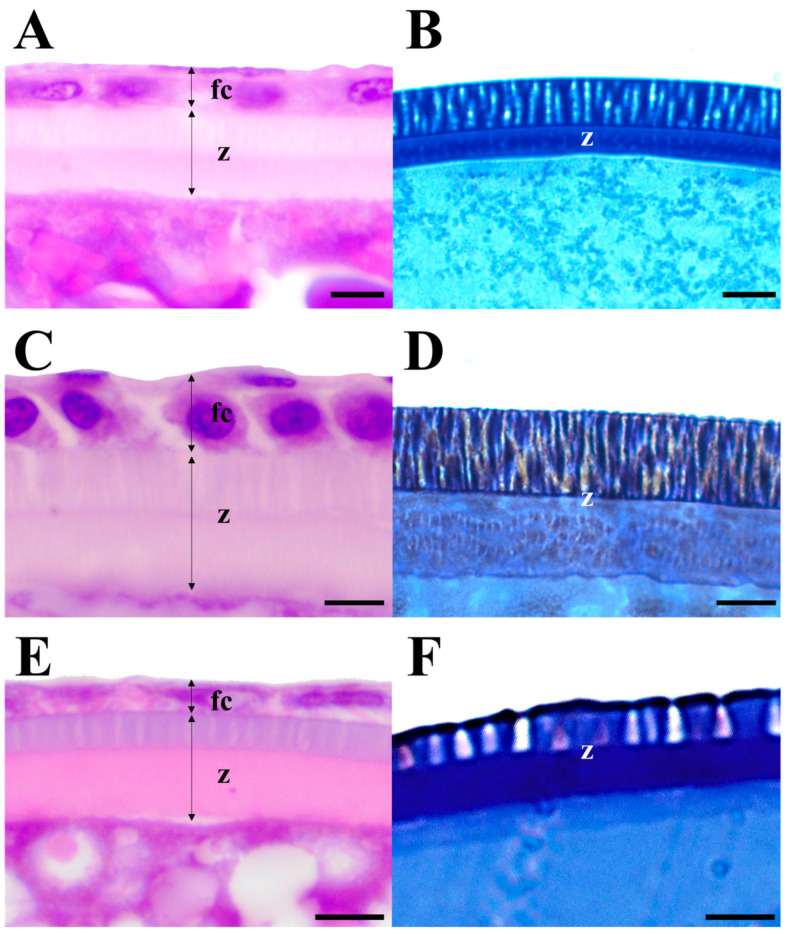
Light micrographs showing the zona radiata in the oocytes of three Cultrinae species based on (**A**,**C**,**E**) Harris’s hematoxylin and eosin staining used non-fertilized eggs in ovary and (**B**,**D**,**F**) 1% toluidine blue staining of fertilized eggs: (**A**,**B**) *Erythroculter erythropterus*; (**C**,**D**) *Culter brevicauda*; (**E**,**F**) *Hemiculter eigenmanni*. fc, follicular layer; z, zona radiata. Scales indicate 5 μm in (**A**–**F**), respectively.

All three species (*E. erythropterus*, *C. brevicauda*, and *H. eigenmanni*) had non-structural forms of egg envelopes on the outer surface; no special structure other than the pore canal was identified on the outer side, and these eggs were non-adherent. The light microscopic analysis of cross-sections of mature eggs revealed striped ZR stained with eosin and 1% toluidine blue. Furthermore, no structure was identified between the outer surface of egg envelopes and the follicular cell layer. In this process, the toluidine-stained oocytes were fertilized eggs; therefore, the follicular cell layers were not identified (Figure 2).

**Figure 3:**
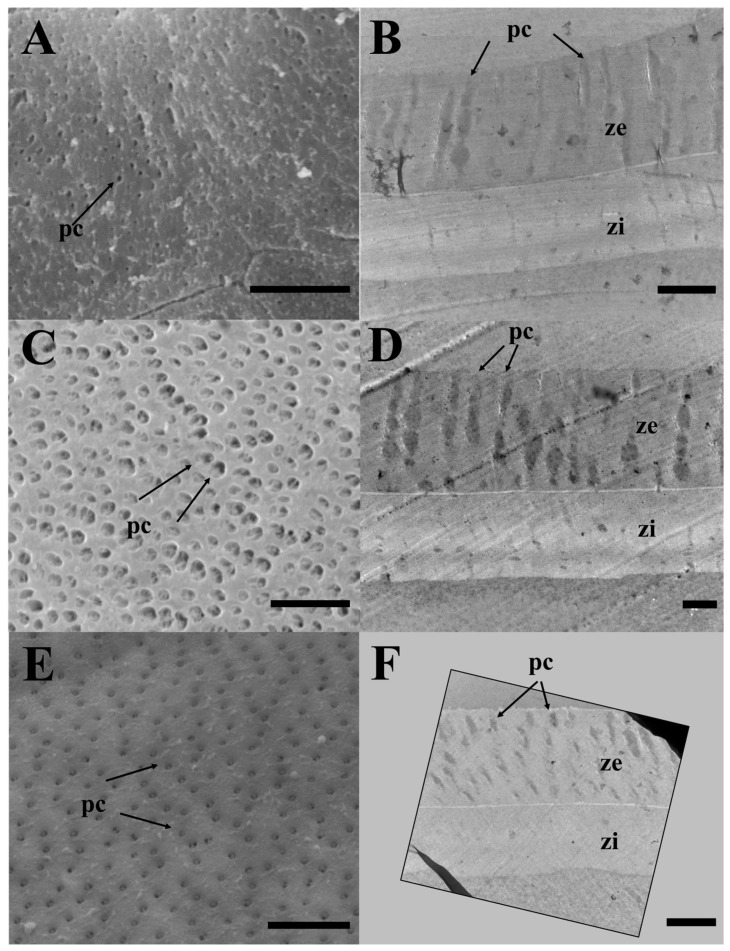
(**A**,**C**,**E**) Scanning and (**B**,**D**,**F**) transmission electron micrographs of zona radiata in three Cultrinae species: (**A**,**B**) *Erythroculter erythropterus*; (**C**,**D**) *Culter brevicauda*; (**E**,**F**) *Hemiculter eigenmanni*. pc, pore canal; ze, zona radiata externa; zi, zona radiata interna. Scales indicate 5 μm in (**A**,**C**,**E**), 2 μm (**B**,**D**,**F**), respectively.

**Figure 4:**
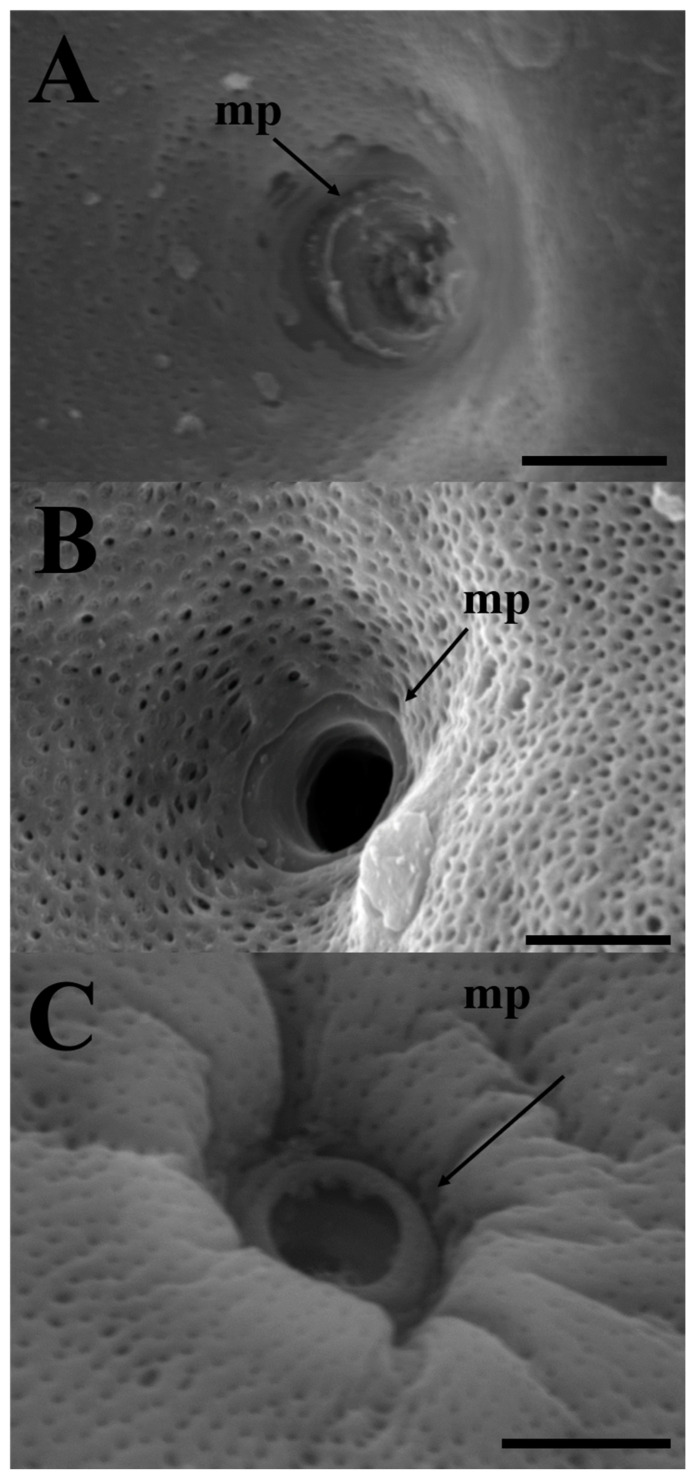
Scanning electron micrographs of micropyles in the three Cultrinae species: (**A**) *Erythroculter erythropterus*; (**B**) *Culter brevicauda*; (**C**) *Hemiculter eigenmanni*. mp, micropyle. Scales indicate 5 μm.

**Figure 5:**
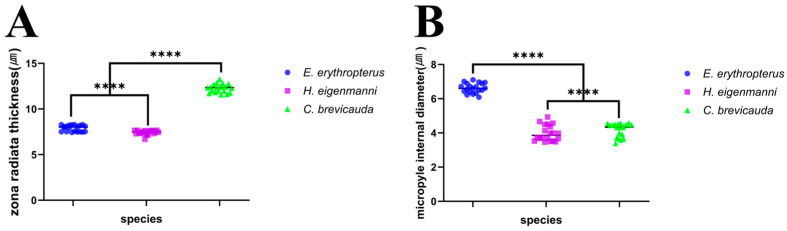
Comparative analysis of zona radiata thickness and diameter of the micropyle of the three Cultrinae: (**A**) zona radiata thickness; (**B**) micropyle internal diameter. **** *p* < 0.0001, when compared with the zona radiata thickness and internal diameter of the micropyle of the three Cultrinae.

The SEM analysis of the surface of mature eggs revealed a large number of pore canals in the egg envelopes—83 for *E. erythropterus*, 75 for *C. brevicauda*, and 58 for *H. eigenmanni* per 10 μm^2^ (Table 1 and Figure 3A,C,E). The TEM analysis of the ZR of the three species was similar to the results of light and SEM; multiple pore canals were identified in the ZR of *E. erythropterus*, *C. brevicauda*, and *H. eigenmanni,* which comprised an outer and inner layer. No other structures were identified on the outer surface of the egg envelopes (Figure 3B,D,F). The thickness of the egg envelopes was measured to be 7.89 ± 0.34 μm in E. *erythropterus*, 12.27 ± 0.46 μm in *C. brevicauda*, and 7.42 ± 0.24 μm in *H. eigenmanni*, and one funnel-shaped micropyle was identified at the tip of each animal pole (Figure 4). Among the three species, *E. erythropterus* had the largest inner diameter of the micropyle (6.62 ± 0.29 μm), followed by *C. brevicauda* (4.19 ± 0.39 μm), and *H. eigenmanni* (3.98 ± 0.46 μm) (Table 1). These results were confirmed by Dunnett’s test and one-way analysis of variance (ANOVA), which showed that the zona radiata thickness and the diameter of the micropyle for each species were significant at a confidence level of *p* < 0.0001. The visualization of these results using GraphPad Prism is shown in Figure 5.

## 4. Discussion

The outer surface of the egg envelope in fish is characterized by a variety of structures, namely non-structural, granular, villous, filamentous, saw-shaped, hillock-shaped, and fence-shaped [1,3]. These structures perform functions such as attachment, hydrostatic regulation, and embryo protection [28,29,30]. The egg envelope structures also play a role in species specificity, demonstrating a close ecological link with the habitat [31,32,33,34]. Park [1] and Choi [35] reported that the ultrastructure of an egg envelope is associated with spawning areas in fish. The non-structural form has been observed in *Misgurnus anguilicaudatus*, *M. mizolepis*, *Lefua costata*, *Gobiobotia nakdongensis*, and *Microphysogobio yaluensis*, which are known to have lentic habitats or spawning areas where the bottom structure comprises mud [36]. Among the Acheilognathinae that spawn in freshwater bivalves, *Acheilognathus lanceolatus*, *A. signifer*, *A. koreensis*, *A. somjinensis*, *A. yamatsutae*, *A. majusculus* and *Sarcocheilichthys nigripinnis morii* of the genus *Sarcocheilichthys* exhibit a non-structural egg envelope, which was found to be favorable for deposition in the gills of bivalves by laying fusiform, pear-shaped, and mono-oval eggs [3]. All three Cultrinae species have bottom structures composed of mud or sand and inhabit lentic waters, which is consistent with previous findings [16]. Additionally, differences in the thickness of the zona radiata of the three species (*E. erythropterus*, *C. brevicauda*, and *H. eigenmanni*) demonstrated species specificity.

In this study, all three species had ZR comprising two layers—an outer and inner layer. The inner layer contains several pore canals composed of microfibers, which are involved in oocyte respiration and nutrient transport [37]. In our study, we identified pore canals in all three species, and although the number varied between species, no other external structures were associated with them. A funnel-shaped micropyle with a larger outer diameter and a smaller inner diameter was observed in all three species of Cultrinae. Although the micropyle morphology of the three species was similar, there were differences observed between the species; *E. erythropterus* demonstrated the largest inner diameter, followed by *C. brevicauda* and *H. eigenmanni*. These micropyles are species-specific and function to prevent multi-fertilization during the spawning season and defend against invasion by sperm from other species [38,39]. Thus, *C. brevicauda* and *H. eigenmanni*, which have similar micropyle sizes, have ecological differences due to different spawning sites and times, and *E. erythropterus* has adapted to its environment, thus having completely different micropyle sizes. Therefore, no cases of hybridization have been reported yet between different species. Some fish have two or more micropyles, which are used as taxonomic characteristics [40,41,42]. The size of the micropyle is known to be closely related to sperm head size [43,44]. The three species in this study live in very large rivers, making it difficult to collect them with conventional nets, and the study would have been difficult to perform without equipment such as SEM and TEM instruments. If we had included the eggs of *Hemiculter leucisculus* in the analysis, we could have expected better results, but it is unfortunate that we could not catch them directly because they live in the Hangang River estuary. However, *H. leucisculus* has a similar habitat, so it is likely that it has a similar egg morphology but with different zona radiata thickness, number of pore canals, and internal size of the micropyle. Finally, we found that the egg structures of *E. erythropterus* and *C. brevicauda*, which are synonyms of scientific names because of their very similar external morphology, are different. These results can be used as a basis for species taxonomy, and we expect that they will be used as a basis for research on aquaculture and the management of invasive species using these data. This can help to manage invasive species by disturbing spawning sites, creating triploids (sterile), or interfering with natural spawning by creating artificial spawning grounds.

## 5. Conclusions

*E. erythropterus* is currently an invasive species in the Nakdonggang River, where it is a top predator that disturbs aquatic ecosystems. *C. brevicauda* has been ecologically declining in the Nakdonggang River and is listed as vulnerable on the IUCN Red List. *H. eigenmanni* is found in all water systems in Korea; therefore, the study of their egg envelope structures is expected to provide basic data for ecology, extermination, and conservation, and it is a relatively large river species that is very difficult to study. This study is, therefore, significant in that it revealed that their egg envelopes are all non-structural; the bottom structure of the stream is primarily composed of mud and sand; they live in slow-flowing water; the micropyle is funnel-shaped but differently sized in all three species; and there are differences in the thickness of the zona radiata and number of pore canals. *E. erythropterus* had the highest number of pore canals required for oxygenation (83), and *H. eigenmanni* had the lowest (58), indicating that even in areas with relatively low oxygen concentrations, *E. erythropterus* had a structure that favored gas exchange without the death of fertilized eggs. *C. brevicauda* had the thickest egg envelopes to protect the eggs from physical impact. Finally, H. eigenmanni, which lived in both *E. erythropterus* and *C. brevicauda* habitats, had the smallest micropyle, indicating that it evolved to form interspecific hybrids. These differences may be used as morphological characters to distinguish between the species of Cultrinae globally, which is currently facing problems due to several species recognized as synonyms and a lack of taxonomic research. These include *E. erythropterus* (named *Chanodichthys erythropterus*, *C. erythropterus*, and *Culterichthys erythropterus*) and *C. brevicauda*. This will additionally serve as a basis for aquaculture research and the management of invasive species of ecosystems in Korea.

## Figures and Tables

**Table 1:** Comparison of the ultrastructure of the egg envelope based on the number of pore canals (10 μm^2^), the thickness of the zona radiata, and the internal diameter of micropyle in the three Cultrinae species (mean ± SD) (range).

Species	Egg Envelope	No. of Pore Canals (*n* = 20)	Zona Radiata Thickness (μm) (*n* = 20)	Micropyle Internal Diameter (μm) (*n* = 20)
*Erythroculter erythropterus*	non-structural form	83	7.89 ± 0.34 (7.43–8.28)	6.62 ± 0.29 (6.09–7.10)
*Culter brevicauda*	non-structural form	75	12.27 ± 0.46 (11.57–13.26)	4.19 ± 0.39 (3.39–4.58)
*Hemiculter eigenmanni*	non-structural form	58	7.42 ± 0.24 (6.69–7.66)	3.98 ± 0.46 (3.47–4.93)

## Data Availability

The data presented in this study are available upon request from the corresponding author.

## References

[1] Park J.Y. (1996). A Morphological Study on the Gonad of the Species in the Family Cobitidae (Pisces: Cypriniformes) from Korea. Ph.D. Dissertation.

[2] Kim S.H., Lee C.H., Ju H.S., Kim H.B., Lee Y.D. (2011). Ultrastructural variations on the micropyle of blacktip grouper, Epinephelus Fasciatus before and after artificial fertilization. Korean J. Microsc..

[3] Choi S.J. (2021). Oogenesis and Ultrastructure of the Egg Envelope in 15 Korean Cyprinid Fish (Pisces, Cypriniformes) Spawning in Bivalves. Ph.D. Dissertation.

[4] Anderson E. (1974). Comparative aspects of the ultrastructure of the female gamete. Int. Rev. Cytol. Suppl..

[5] Flegler C. (1977). Electron microscopic studies on the development of the chorion of the viviparous teleost Dermogenys pusillus (Hemirhamphidae). Cell Tissue Res..

[6] Kobayashi W., Yamamoto T.S. (1981). Fine structure of micropylar apparatus of the chum salmon egg, with a discussion of the mechanism for blocking polyspermy. J. Exp. Zool..

[7] Cotelli F., Andronico F., Bassi R., Brivio M., Ceccagno C., Denis-Donini S., La Rosa M.L., Lamia Donin C.L. (1986). Studies on the composition, structure and differentiation of fish egg chorion. Cell Biol. Int. Rep..

[8] Li Y.H., Wu C.C., Yang J.S. (2000). Comparative ultrastructural studies of the zona radiata of marine fish eggs in three genera in Perciformes. J. Fish Biol..

[9] Wourms J.P. (1976). Annual fish oogenesis: Differentiation of the mature oocyte and formation of the primary envelope. Devl. Bio..

[10] Ivankov V.N., Kurdyayeva V.P. (1973). Systematic differences and the ecological importance of the membranes in fish eggs. J. Ichthyol..

[11] Kim D.H., Reu D.S., Deung Y.G. (1996). A comparative study on the ultrastructures of the egg envelope in fertilized eggs of fishes, Characidae, three species. Korean J. Electron. Microsc..

[12] Riehl R., Patzner R.A. (1998). Minireview: The modes of egg attachments in teleost fishes. Ital. J. Zool..

[13] Dai Y.G., Yang J.X. (2003). Phylogeny and Zoogeography of the Cyprinid Hemicultrine Group (Cyprinidae: Cultrinae). Zool. Stud..

[14] Dai Y.G., Yang J.X., Chen Y.R. (2005). Phylogeny and zoogeography of the subfamily Cultrinae (Cyprinidae). Acta Zootaxonomica Sin..

[15] Nelson J.S., Grande T.C., Wilson M.V.H. (2016). Fishes of the World.

[16] Kim I.S., Park J.Y. (2002). Freshwater Fishes of Korea.

[17] Chae B.S., Song H.B., Park J.Y. (2019). A Field Guide to the Freshwater Fishes of Korea.

[18] ME (Ministry of Environment) (2022). Spatial Distribution, Population Growth, and the Efficient Management Introduced Fish Species (Erythroculter erythropterus) in Nakdong River.

[19] Coad B.W., Hussain N.A. (2007). First record of the exotic species Hemiculter leucisculus (Actinopterygii: Cyprinidae) in Iraq. Zool. Middle East.

[20] Jouladeh-Roudbar A., Vatandoust S., Eagderi S., Jafari-Kenari S., Mousavi-Sabet H. (2015). Freshwater fishes of Iran; an updated checklist. Aquac. Aqar. Conserv. Legis.

[21] Mousavi-Sabet H., Heidari A., Salehi M. (2019). Reproductive biology of the invasive sharpbelly, Hemiculter leucisculus (Basilewsky, 1855), from the southern Caspian Sea basin. Iran. J. Ichthyol..

[22] ME (Ministry of Environment) (2016). Study on the Management Method of Invasive Freshwater Fishes.

[23] Chen W., Zhong Z., Dai W., Fan Q., He S. (2017). Phylogeographic structure, cryptic speciation and demographic history of the sharpbelly (Hemiculter leucisculus), a freshwater habitat generalist from southern China. BMC Evol. Biol..

[24] Wang L., Zhu L., Tang K., Liu M., Xue X., Wang G., Wang Z. (1855). Population genetic structure of sharpbelly Hemiculter leucisculus and morphological diversification along climate gradients in China. Ecol. Evol..

[25] Gu Q., Zhong H., Sun Y., Yuan H., Li S., Shen Z., Wen M. (2022). Reanalysis on Phylogeographic Pattern of Sharpbelly Hemiculter leucisculus (Cyprinidae: Cultrinae) in China: A Review and the Implications for Conservation. Front. Ecol. Evol..

[26] Cummins K.W. (1962). An evolution of some techniques for the collection and analysis of benthic samples with special emphasis on lotic waters. Am. Midl. Nat..

[27] Gurr E. (1956). A Practical Manual of Medical and Biological Staining Techniques.

[28] Laale H.W. (1980). The perivitelline space and egg envelopes of bony fishes, a review. Copeia.

[29] Hiromi O. (1984). Electron microscopic study on adhesive material of pacific herring (Clupea pallasi) eggs. Jpn J. Ichthyol..

[30] Rizzo E., Sato Y., Barreto B.P., Godinho H.P. (2002). Adhesiveness and surface patterns of eggs in neotropical freshwater teleosts. J. Fish Biol..

[31] Stehr C.M., Hawkes J.W. (1979). The comparative ultrastructure of the egg membrane and associated pore structure in the starry flounder, Platichthys stellatus (Pallas), and pink salmon, Oncorhynchus gorbuscha (Walbaum). Cell Tissue Res..

[32] Groot E.P., Alderdice D.F. (1985). Fine structure of the external egg membranes of five species of Pacific salmon and steelhead trout. Can. J. Zool..

[33] Berrada-Rkhami O., Gabrion C. (1990). The fine structure of the embryonic envelopes before and after hatching in bothriocephalids: Physiological and ecological significance. Parasitol. Res..

[34] Hirai A. (1993). Fine structure of the egg membrane in four species of Pleuronectinae. Jpn J. Ichthyol..

[35] Choi W.S. (2015). The oogenesis and ultrastructure of egg envelope in 3 species of genus Gobiobotia (Pisces: Cyprinidae) from Korea. Master’s Thesis.

[36] Kim J.G., Reu D.S., Park J.Y. (2017). Oogenesis of Microphysogobio yaluensis (Pisces, Cyprinidae) in the Korean endemic species. Korean J. Ichthyol..

[37] Nagahama Y. (1983). The Functional Morphology of Teleost Gonads. Fish Physiology.

[38] Grierson J.P., Neville A.C. (1981). Helicoidal architecture of fish eggshell. Cell Tissue Res..

[39] Cameron I.L., Hunter K.E. (1984). Regulation of the permeability of the medaka fish embryo chorion by exogeneous sodium and calcium ions. J. Exp. Zool..

[40] Chen K.C., Shao K.T., Yang J.S. (1999). Using micropylar ultrastructure for species identification and phylogenetic inference among four species of Sparidae. J. Fish Biol..

[41] Morisawa S. (1999). Fine structure of micropylar region during late oogenesis in eggs of the hagfish Eptatretus burgeri (Agnatha). Dev. Growth Differ..

[42] Debus L., Winkler M., Billard R. (2002). Structure of micropyle surface on oocytes and caviar grains in Sturgeons. Int. Rev. Hydrobiol..

[43] Hart N.H. (1990). Fertilization in teleost fishes: Mechanisms of sperm-egg interactions. Int. Rev. Cytol..

[44] Linhart O., Kudo S. (1997). Surface structure of paddlefish eggs before and after fertilization. J. Fish Biol..

